# A Novel Role for Stat1 in Phagosome Acidification and Natural Host Resistance to Intracellular Infection by *Leishmania major*

**DOI:** 10.1371/journal.ppat.1000381

**Published:** 2009-04-17

**Authors:** Gerald F. Späth, Paul Schlesinger, Robert Schreiber, Stephen M. Beverley

**Affiliations:** 1 Department of Molecular Microbiology, Washington University Medical School, St. Louis, Missouri, United States of America; 2 Department of Parasitology and Mycology, Laboratory of Parasite Virulence, Institut Pasteur, Paris, France; 3 Institut National de la Santé et de la Recherche Médicale (INSERM) AVENIR, Paris, France; 4 Department of Cell Biology, Washington University Medical School, St. Louis, Missouri, United States of America; 5 Department of Pathology and Immunology, Washington University Medical School, St. Louis, Missouri, United States of America; University of Wisconsin-Madison, United States of America

## Abstract

Intracellular parasites of the genus *Leishmania* generate severe diseases in humans, which are associated with a failure of the infected host to induce a protective interferon γ (IFNγ)-mediated immune response. We tested the role of the JAK/STAT1 signaling pathway in *Leishmania* pathogenesis by utilizing knockout mice lacking the signal transducer and activator of transcription 1 (Stat1) and derived macrophages. Unexpectedly, infection of Stat1-deficient macrophages *in vitro* with promastigotes from *Leishmania major* and attenuated *LPG1* knockout mutants (*lpg* ^−^) specifically lacking lipophosphoglycan (LPG) resulted in a twofold increased intracellular growth, which was independent of IFNγ and associated with a substantial increase in phagosomal pH. Phagosomes in Stat1−/− macrophages showed normal maturation as judged by the accumulation of the lysosomal marker protein rab7, and provided normal vATPase activity, but were defective in the anion conductive pathway required for full vesicular acidification. Our results suggest a role of acidic pH in the control of intracellular *Leishmania* growth early during infection and identify for the first time an unexpected role of Stat1 in natural anti-microbial resistance independent from its function as IFNγ-induced signal transducer. This novel Stat1 function may have important implications to studies of other pathogens, as the acidic phagolysosomal pH plays an important role in antigen processing and the uncoating process of many viruses.

## Author Summary

Protozoan parasites of the genus *Leishmania* generate a variety of pathologies, collectively termed leishmaniasis, which afflict millions of people worldwide. *Leishmania* is transmitted during the blood meal of infested sand flies that inoculate highly infective metacyclic promastigotes into the mammalian host. Following uptake by host macrophages, metacyclics differentiate into the amastigote form that replicates inside the acidified phago-lysosome of the host cell. The cytokine interferon-γ activates infected macrophages to kill intracellular *Leishmania* through the production of nitric oxide. This process is mediated through Stat 1, a cytosolic transcription factor that translocates into the nucleus in response to the cytokine, where it induces a pleiotropic anti-microbial response. By utilizing Stat1-deficient macrophages we found evidence for a novel interferon-γ-independent physiological function of Stat1 in acidification of the host cell phago-lysosome. Stat1-deficient macrophages showed higher phago-lysosomal pH and increased susceptibility to *Leishmania* infection, which was linked to a defect in cellular chloride channel function. Vesicular pH and acidification are important factors affecting the infective cycle of bacterial and protozoan pathogens, and the uncoating process during viral entry. Thus, the role of cytokine-independent Stat1 functions in innate anti-microbial resistance may have a greater impact on host-pathogen interactions than previously appreciated.

## Introduction

Protozoan parasites of the genus *Leishmania* generate a variety of pathologies collectively termed leishmaniasis that afflict millions of people worldwide [1]. Depending on parasite species and host immune response, the pathologies range from mild cutaneous, self-healing lesions generated by *L. major*, to the fatal visceral disease caused by *L. donovani*. *Leishmania* is transmitted during blood feeding of infected sand flies, which inoculate highly infective metacyclic promastigotes into the mammalian host [2]. Following uptake by host macrophages, metacyclics differentiate into the amastigote form that replicates inside the fully acidified phago-lysosome of the host cell. From this site the parasite modulates the response of the host cell and immune system [3],[4].

Release of IL-4 and IL-10 by infected macrophages and accessory immune cells establishes a TH2 response permissive for parasite growth and responsible for acute disease with fatal outcome in immuno-compromised individuals and susceptible BALB/c mice [5],[6]. In contrast, immuno-competent individuals and genetically resistant mouse strains, including C57BL/6, B10, and SV129 [7], mount a Th1 response and are able to contain parasite growth during later stages of the infection by the production of IL-12 that entails development and expansion of histocompatibility complex MHC class II-restricted Th1 cells [8],[9]. Interferon γ (IFNγ) secreted by these cells elicits a pleiotropic anti-microbial response in macrophages that is transduced by the inducible transcription factor Stat1 [10],[11], a cytosolic latent transcription factor that forms dimers and translocates into the nucleus following tyrosine phosphorylation by Janus family tyrosine kinases [12]. There, Stat1 induces expression of iNOS and pro-inflammatory cytokines including IL-12, TNFα, and IL1β, which are required for resistance to various parasitic, bacterial and viral pathogens.

A role for Stat1 distinct from its function as inducible transcription factor has been suggested [13]. Stat1-deficient fibroblast cell lines showed reduced expression of the low molecular mass proteins LMP-1 and LMP-2 [14],[15], and the caspases ICE, Cpp32 and Ich-1, associating constitutive Stat1 activity with antigen processing and apoptosis [14]. Here we report evidence for a novel physiological function of Stat1 in phagosomal acidification, which was independent from IFNγ and its activity through the well known roles of this important transcription factor in immune function. The selective defect of Stat1−/− cells allowed us for the first time to test the role of phagosomal pH on *Leishmania* survival *in situ*.

## Results

### Stat1 is required for anti-leishmanial resistance in mouse and macrophage infection

**Figure 1:**
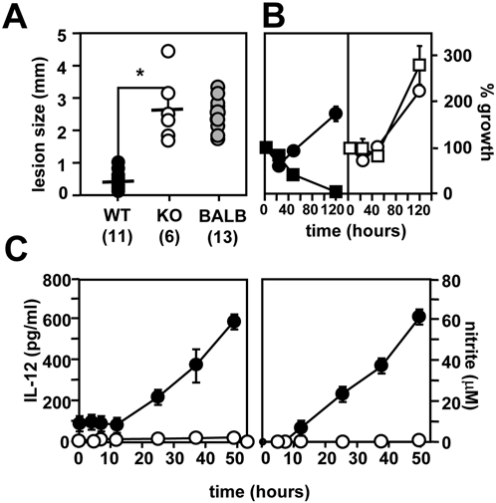
Stat1-deficient mice are susceptible to *Leishmania* infection. (A) Footpad analysis. 10^6^ *L. major* promastigotes from stationary culture were inoculated subcutaneously into the hind footpad of SV129 control (closed circles), SV129 Stat1−/− (open circles), and susceptible BALB/c mice (gray circles), and lesion size was followed for 90 days post-infection. The graph represents the footpad swelling at day 70 post-infection after lesions resolved in the resistant SV129 controls (number of infected animals per group is shown in brackets). *, p = 2e-6. (B) Macrophage infection. Stat1-deficient (open symbols) and SV129 wild-type PEMs (closed symbols) treated with 100 U/ml IFNγ and 100 ng/ml LPS for 12 h (squares) and untreated controls (circles) were infected with complement-opsonized stationary promastigotes and the number of intracellular parasites per 100 macrophages was determined microscopically. Three independent experiments were performed and one representative triplicate experiment is shown. (C) Macrophage activation. 10^4^ peritoneal exudate macrophages (PEM) of SV129 control (closed circles) and Stat1−/− (open circles) were seeded into micro-titer plates and treated with 100 U/ml IFNγ and 100 ng/ml LPS. Levels of IL-12p70 (left panel) and nitrite (right panel) were determined in the supernatants at the time points indicated. The bars represent the standard deviation of one triplicate experiment.

Groups of Stat1-deficient mice and SV129 isogenic controls, or susceptible BALB/c mice, were inoculated with 10^6^ infective *L. major* promastigotes, and the ability to resolve the infection was assessed during 12 weeks post-infection. In resistant SV129 mice, the parasites elicited a transient lesion, which was completely resolved in all 11 animals 70 days after the infection (Figure 1A). In contrast, SV129 Stat1−/− mice were unable to control the infection and showed progressive lesion development similar to susceptible BALB/c mice with ultimately fatal outcome , as previously shown [16]. We further investigated this defect by *in vitro* infection of peritoneal exudate macrophages (PEM).

Intracellular *Leishmania* growth was assessed in untreated and LPS/IFNγ-activated PEMs from wild-type and Stat1-deficient mice by nuclear staining and fluorescence microscopy [17]. Parasites showed robust intracellular growth in untreated control PEMs, which was completely abolished in activated cells (Figure 1B, left panel). In contrast, Stat1−/− PEMs were highly permissive for intracellular *Leishmania* growth, even in LPS/IFNγ-treated cells (Figure 1B, right panel).

In immunocompetent hosts, *L. major* infection is controlled by the induction of leishmanicidal NO in response to IFNγ-producing Th1 cells, which in turn differentiate in an IL12-dependent manner. IFNγ/LPS-treated PEMs from Stat1−/− mice were unable to produce IL12 or nitric oxide, while robust levels were detected in the supernatants of treated controls (Figure 1C). Together these data confirm the crucial role of IFNγ in controlling *Leishmania* infection through Stat1-mediated cytokine and NO production, and further sustain the importance of macrophage activation in anti-leishmanial resistance.

### Increased *Leishmania* survival in Stat1-deficient PEMs

**Figure 2:**
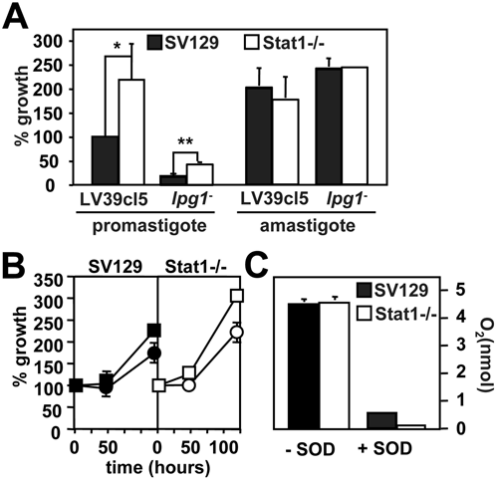
Stat1-deficient macrophages are more permissive for *Leishmania* infection. (A and B) Macrophage infection. (A) Wild-type and *lpg1* ^−^ promastigotes (left panel) or lesion-derived amastigotes (right panel) were opsonized with C3b and incubated with peritoneal macrophages derived from SV129 control and Stat1−/− mice. The number of intracellular parasites was estimated by nuclear staining and fluorescence microscopy throughout the infection period. Parasite survival at day 5 (for promastigotes) and at day 2 (for amastigotes) was normalized to the initial infection efficiencies at 2 h post-infection. Three independent experiments were performed and the bars show the standard deviations of one representative triplicate experiment. *, p = 0.12; **, p = 0.01. In (B), parasite survival was determined in untreated (circles) and NMMA-treated PEMs (squares). (C) *Superoxide determination*. Superoxide production of confluent monolayers of SV129 control and Stat1−/− peritoneal macrophages was determined by ferrycytochrome c reduction assay following incubation with zymosan in the presence (+SOD) and the absence (−SOD) of 100 ng/ml superoxide dismutase. The optical density of the supernatants was determined spectrophotometrically using supernatants from untreated cells as blank.

During the macrophage infection studies, we consistently observed a trend towards increased intracellular parasite growth in naïve Stat1−/− PEMs when compared to wild-type controls. We quantified this unexpected effect following infection with promastigotes form wild-type *L. major* and mutant lacking the abundant surface lipophosphoglycan through inactivation of the *LPG1* gene [17]. As expected from previous results [17], survival of *lpg* ^−^ promastigotes in SV129 PEM was reduced by 75% (Figure 2A). A similar reduction was observed in Stat1−/− PEMs confirming our previous results that intracellular elimination of *lpg* ^−^ is independent from IFNγ-mediated effects [18]. Surprisingly, even though the infections were performed in the absence of IFNγ and thus under conditions where Stat1 should be inactive, survival of both wild-type and *lpg* ^−^ promastigotes was increased in Stat1−/− PEMs by more than twofold (Figure 2A). In contrast, lesion-derived wild-type amastigotes survived equally well in Stat1−/− macrophages and controls regardless of host or parasite phenotype (Figure 2A, right panel).

We first tested if increased promastigote survival in Stat1−/− cells resulted from their failure to produce leishmanicidal NO (see Figure 1C, right panel). Stat1−/− PEMs and controls were treated with the NO-inhibitor NMMA and intracellular parasite survival was determined as described above and compared to untreated controls. Again, Stat1−/− PEMs were more permissive for intracellular *Leishmania* growth compared to the wild-type (WT) control, even in the presence of NMMA (Figure 2B). Both control and Stat1−/− PEMs produced similar amounts of superoxide during phagocytosis, which was strongly reduced upon treatment of the supernatants with superoxide dismutase (Figure 2C). These data rule out a role for reactive nitrogen or oxygen radicals (or the absence thereof) in increased Stat1−/− *Leishmania* survival.

### A selective defect of Stat1−/− PEMs in phagosomal acidification

**Figure 3:**
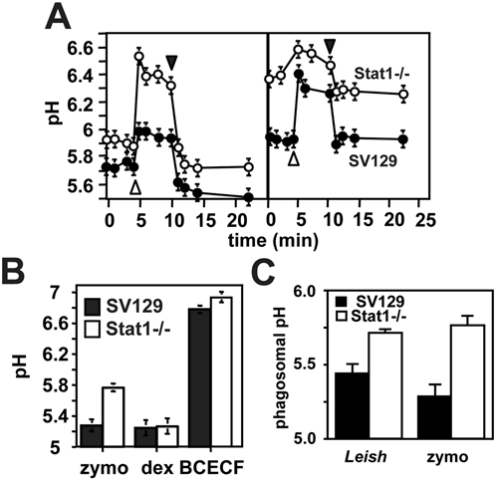
Stat1−/− phagosomes show a selective defect in phagosomal acidification. (A) Phagosomal pH-measurement. Monolayers of untreated (left panel) or LPS/IFNγ treated (right panel) SV129 control and Stat1−/− PEMs were established on 1 by 2.5 cm glass slides and pH of phagosomes containing FITC-labeled zymosan was determined by ratio-fluorescence determination. Calibration for fluorescence was done in situ. The fraction of particles bound to and remaining on the cell surface was controlled for and found to be negligible by measurement of the response to alterations in the pH of the external buffer. Each experiment was performed in duplicate and repeated at least three times. The graph shows a representative time course measurement of phagosome alkalinization upon addition of 10 µM NH_4_Cl (open arrow head), equilibration and removal (closed arrow head). (B) and (C) Measurement of intracellular pH. (B) Monolayers of SV129 control and Stat1−/− PEMs were incubated for 30 min with 10 µM BCECF-AM, overnight with 2.5 mg/ml FITC-dextran, or 2 h with FITC-labeled zymosan, and cytoplasmic, lysosomal, or phagosomal pH were assessed by ratio-fluorescence determination. The bars represent the standard deviation of three independent duplicate experiments. (C) Monolayers of SV129 control and Stat1−/− PEMs were infected for 2 h with FITC-labeled axenic *L. donovani* amastigotes (Leish) or incubated with FITC-labled zymosan (zymo), and phagosomal pH were assessed by ratio-fluorescence determination.

We followed the maturation of phagosomes into acidic phago-lysosomes by fluorescence ratio determination. Monolayers of untreated or LPS/IFNγ treated SV129 control and Stat1−/− PEMs were incubated with zymosan-FITC and intra-vesicular pH was determined spectrophotometrically by establishing the ratio of pH-independent to pH-dependent florescence at 450 and 495 nm respectively. Following phagosome alkalinization in the presence of 10 µM NH_4_Cl (open arrow head), equilibration and removal of the base (closed arrow head), phagosomes of untreated control PEMs equilibrated at an intra-vesicular pH of 5.3 consistent with previous findings (Figure 3A) [19],[20]. In contrast, phagosomes of untreated Stat1-deficient cells failed to fully acidify and showed a substantial increase of 0.6 units in intra-vesicular pH to pH 5.9 (Figure 3A, left panel). Treatment of the cells with LPS/IFNγ substantially inhibited acidification of WT and Stat1-deficient phagosomes, which equilibrated at pH 5.9 and 6.3 respectively (Figure 3A, right panel). Thus macrophage activation results in increased phagolysosomal pH thereby ruling out the possibility that residual IFNγ production in WT PEMs may contribute to the observed difference in phagosomal acidification. We analyzed cytoplasmic and lysosomal pH in cells incubated for 12 h in DMEM with 10 µM BCECF-AM and 2.5 mg/ml of dextran-FITC respectively (Figure 3B). Both control and Stat1-deficient cells provided a neutral cytoplasmic pH of 6.8 to 6.9 and an acidic lysosomal pH of 5.2. Addition of increasing concentrations of NH_4_Cl (10, 20 and 50 µM, not shown) allowed us to determine a buffering capacity of 54±8 mmoles/mpH for either macrophage [21].We next established that the pH defect of Stat1−/− PEMs occurs also during *Leishmania* infection ,using FITC surface-labeled *Leishmania* and intra-vesicular fluorescence-ratio measurement. We used axenic amastigotes from *L. donovani*, which do not express LPG and thus eliminate concerns regarding the release of labeled LPG into other cell compartments and its effect on phago-lysosomal fusion [22],[23]. Similar to the zymosan control, Stat1−/− phagosomes do not fully acidify following uptake of labeled amastigotes and equilibrate at 0.3 pH units higher than controls (Figure 3C).

Thus, Stat1−/− PEMs show a selective defect in phagosomal acidification independent from lysosomal pH, which may enhance intracellular parasite survival.

### Normal recruitment of the lysosmal marker Rab7 in Stat1−/− PEMs

**Figure 4:**
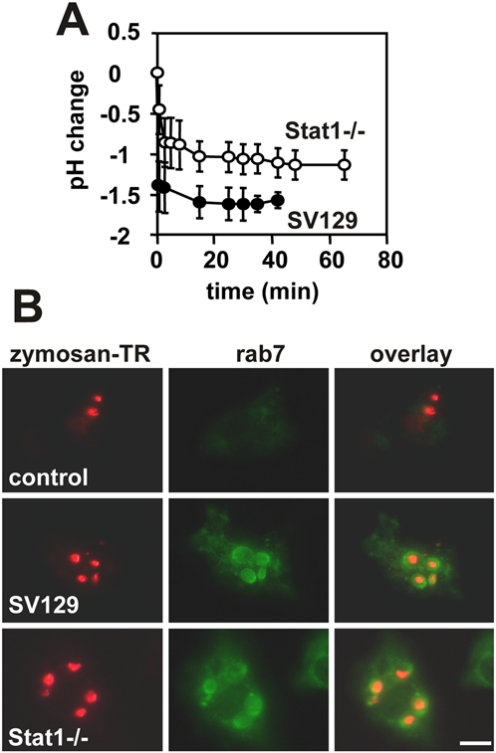
Stat1-deficient PEMs are normal in phagosome maturation. (A) Kinetics of phago-lysosomal fusion. Monolayers of SV129 control and Stat1−/− PEMs were incubated with FITC-labeled zymosan at 4°C for 20 min. Phagosomal pH was monitored by ratio-fluorescence determination during synchronized phagosome formation and acidification at 37°C. (B) Indirect immunofluorescence analysis. Control and Stat1−/− PEMs were incubated on ice with TexasRed-labeled zymosan for 20 min and synchronous uptake was established by temperature shift to 37°C. Cells were fixed at time points 0, 30 (shown here), 60 and 90 min after uptake, and subcellular localization of the lysosomal marker rab7 was assessed by indirect immunofluorescence. Control cells were stained with the FITC-conjugated secondary antibody alone. The bar corresponds to 10 µm.

Maturation of phagosomes into an acidic, hydrolase-rich compartment depends upon interactions with the endocytic network and the fusion with late endosomes or lysosomes [24]. Thus partial acidification of phagosomes in Stat1-deficient macrophages may result from a failure to interact with these acidic organelles. We established a detailed kinetics of phagosomal acidification by fluorescence ratio measurement. Control and Stat1−/− PEMs were incubated with zymosan-FITC for 20 min at 4°C and intra-vesicular pH was determined during synchronous uptake induced by temperature shift to 37°C. PEMs from both control and deficient mice provided similar kinetics of phagosome acidification during the first minutes after zymosan uptake, however Stat1−/− phagosomes equilibrated shortly after at 0.5 pH units above the pH attained in control PEMs (Figure 4A). Phagosome maturation was further studied by accumulation of the late endosomal marker protein rab7 [25]. During the synchronous uptake of Texas Red-labeled zymosan, rab7 was absent in early phagosomes of control and Stat1-deficient PEMs (up to 20 min post-incubation) and detected in perinuclear vesicular compartments (data not shown). Rab7 was first detected in phagosomes of both control and Stat1−/− PEMs 30 min after zymosan uptake and was maintained thereafter for the rest of the incubation period (Figure 4B). Thus the defect in phagosomal acidification is independent from lysosomal fusion as judged by the recruitment of the lysosomal marker Rab7.

### Limited proton transporting activity in Stat1−/− PEMs

Vesicle acidification is achieved by the combined action of an electrogenic H^+^-ATPase, which pumps protons into the lumen, and a chloride-channel that short-circuits the electrical potential across the membrane, allowing proton transport further to continue. We tested if a defect in one of these activities accounts for the elevated phagosomal pH in Stat1-deficient macrophages.

**Figure 5:**
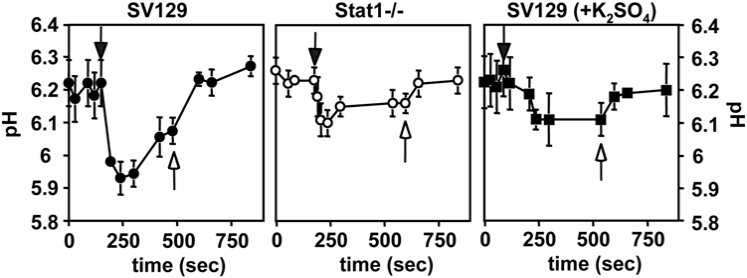
pH measurement on isolated phagosomes. Intra-vesicular pH of phagosomes containing FITC-labeled zymosan was determined in untreated control preparations (left panel), untreated preparations from Stat1−/− BMMs (middle panel), and control preparations treated with 93 mM K_2_SO_4_ (right panel). Acidification was initiated by addition of 2.5 mM ATP and 1 mM MgSO_4_ (closed arrow). Subsequent addition of 20 µM NH_4_Cl increased the pH of all phagosome preparations (open arrow). The addition of 10 µM nigericin increased the pH to 7 (not shown). Each time course was done in triplicate with the standard deviation indicated by the error bars.

Phagosomes containing FITC-conjugated zymosan were isolated from control and Stat1-deficient bone marrow-derived macrophages (BMM), diluted into the reaction mixture containing ATP and reactions were started by the addition of MgSO_4_ (Figure 5, closed arrows). Phagosomes from control mice showed a rapid but transient decrease in vesicular pH by 0.3 pH units to 5.95 (s.d. 0.04) during the first minute after MgSO_4_ addition (Figure 5, left panel). Phagosomes from Stat1−/− BMMs were able to initiate phagosome acidification (Figure 5, middle panel) but showed a pH decrease of only 0.15 pH units to 6.13 (s.d.0.04). This acidification profile indicates the presence of a functional H^+^-ATPase that provides limited activity most likely due to a defect in charge neutralization compared to the control (p<0.002 for the difference observed one minute after ATP addition). This hypothesis was further sustained in K_2_SO_4_-treated control preparations. Replacement of chloride with impermeant anion sulfate eliminates the charge neutralization normally conferred by the chloride channel, a treatment that resulted in partial acidification of Stat1+/+-preparations similar to the one observed in Stat1−/− preparations (Figure 5, right panel).

### Stat1-deficient phagosomes are defective in charge-neutralization

**Figure 6:**
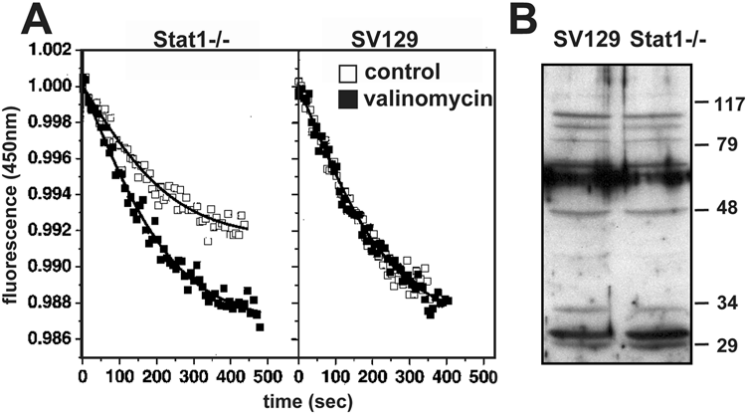
Analysis of vesicle acidification by acridine orange fluorescent quenching. (A) Membrane vesicles derived from phagosomes of Stat1-deficient BMMs and control were diluted into the appropriate reaction mixture containing 2.5 mM ATP, and reactions were started by the addition of 1 mM MgSO_4_ either in the absence (control, open squares) or in the presence of 0.1 µM valinomycin (closed squares). All experiments were corrected for base-line drift (<5% of total fluorescence). (B) Crude cell extracts were subjected to Western Blot analysis with anti-chloride channel antibody AB656.

We tested the charge neutralizing activity in reconstituted vesicles from membrane preparations of control and Stat1-deficient BMMs. Mg^2+^-ATP-dependent proton transport was determined following quenching of acridine orange fluorescence, a weak base that accumulates in acidic compartments and shows a pH-dependent decrease in fluorescence during vesicle acidification [26]. Vesicles derived from both cell types were able to initiated acidification upon addition of MgSO_4_ in the presence of ATP, however vesicles derived from Stat1-deficient cells acidified only partially when compared to the control (Figure 6A, left panel). Acidification was restored to normal levels in these preparations in the presence of valinomycin, a potassium ionophore that eliminates the chloride-dependence of acidification by collapsing the potential generated by the proton pump. These data show that Stat1-deficient macrophages are defective in charge neutralization most likely due to a chloride channel dysfunction [19],[27]. Western Blot analysis of crude and phagosomal extracts (Figure 6B and data not shown) with polyclonal antibody AB656 [26] revealed similar levels in expression of the chloride channel family members detected by this antibody in control and Stat1-deficient preparations, suggesting that the defect in the mutant cells may be linked to a difference in activity rather than expression of chloride channel proteins, or results from the absence of chloride channel species not detected by this antiserum.

### *Leishmania* survival in Stat1-deficient macrophages is independent from phago-lysosomal fusion

Immediately following phagocytosis by host macrophages, *Leishmania* promastigotes transiently inhibit phagolysosomal fusion, a process mediated by LPG [18],[23]. We recently showed that this delay in phagosome maturation did not alter survival of either wild-type or *lpg* ^−^parasites [18]. The Stat1−/− PEMs allowed us for the first time to test the effect of phagosomal pH on parasite survival *in situ*, providing a second perspective on our previous findings.

**Figure 7:**
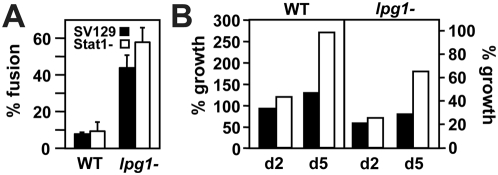
Phago-lysosomal fusion does not affect *Leishmania major* survival. (A) Phago-lysosomal fusion was quantified by labeling of parasitophorous vacuoles with FITC-dextran, previously loaded into the lysosomal compartment (Methods). (B) PEM infection was performed as described in legend of Figure 1B. Experiment was done in duplicate and mean values are shown.

Control and Stat1−/− PEMs previously labeled with dextran-FITC were infected synchronously with either wild-type or *lpg1* ^−^ *Leishmania* and fusogenic phagosomes were identified by florescence microscopy 3 h later as described [18]. As previously shown, wild-type parasites reside in non-fusogenic phagosomes (Figure 7A). As expected form the absence of LPG, phagosomes containing *lpg1* ^−^ parasites were highly fusogenic [23],[28]. Significantly, the exposure to lysosomal content in SV129 control and Stat1−/− cells had no effect on parasite survival during the first 2 days post-infection, when *Leishmania*-containing phagosomes are generally fully acidified (Figure 7B). In contrast, parasite numbers showed a substantial increase in Stat1−/− PEMs between day 2 and day 5 post-infection, when amastigote differentiation was completed and intracellular growth initiated. Together these data suggest that Stat1−/− PEMs show normal fusogenic properties during *Leishmania* infection. Additionally, the fact that LPG-deficient parasites show no difference in intracellular survival during the first 48 h in WT and Stat1−/− macrophages, despite the significant difference in their phagolysosomal pH, further supports the conclusion that killing of the LPG-deficient mutant is independent of phagosome acidification.

## Discussion

The inducible transcription factor Stat1 transmits the immune-protective effects of IFNγ during viral, bacterial and parasitic infections [10],[11],[16],[29],[30]. Previously, a constitutive activity of Stat1 has been identified that regulates target gene expression in an IFNγ-independent manner [14],[15]. However, the significance of this pathway on host immunity and its impact on the interpretation of studies performed in Stat1-deficient animals had not been studied. Here we describe for the first time a novel function of constitutive Stat1 in modulation of phagosomal acidification.

Fusion of phagosomes with hydrolase-rich, acidic compartments including lysosomes and endosomes [31] establishes a hostile environment to potential pathogens as well as comprising a key compartment for antigen presentation [32]–[34]. The relevance of lysosomal degradation in anti-leishmanial resistance has been genetically defined by studies of the natural-resistance-associated macrophage protein, NRAMP1 [35], a transmembrane phosphoglycoprotein which confers natural resistance to a variety of intracellular pathogens [36] by regulating the intra-phagosomal pH [37]. By utilizing *in vitro Leishmania* infection assays we identified a selective defect in phagosomal acidification in Stat1-deficient macrophages (Figure 3), which resulted in a twofold increase of intracellular parasite survival during a 5 days infection period (Figure 2).

The selective Stat1−/− defect in acidification allowed us to investigate *in situ* the role of phagosomal pH on *Leishmania* survival and growth. A potential role for acidic pH in anti-leishmanial resistance has been put forward by Desjardins and co-workers based on the observation that promastigotes reside transiently in non-fusogenic phagosomes [23],[28],[38]. This effect is mediated by the major surface glycoconjugate LPG, which is released from the parasite surface into the host cell cytoplasm, where it interferes with vesicular fusion [22],[39]. Hence, *Leishmania* may have evolved an intracellular survival strategy reminiscent to other pathogens, including *Toxoplasma* [40], *Legionella* [41] and *Mycobacteria* [20],[36], all of which avoid contact with the lysosomal content.

Increased survival of intracellular *L. major* in Stat1-deficient host cells seems to support a role for phagosomal acidification in anti-leishmanial resistance. However, we and others have provided previously compelling evidence that *Leishmania* promastigotes are perfectly well adapted for survival in acidic environments. Promastigotes grow normally at pH 5.5 [42], and their surface glycocalyx confers resistance to lysosomal hydrolases in insect and vertebrate hosts [43]–[46]. We previously showed that intracellular survival of attenuated *lpg* ^−^ mutants was restored to wild-type levels in oxidant-deficient *phox*−/− host cells, although extensive fusion of parasite-containing phagosomes with host cell lysosomes occurred [18]. Here we confirmed these data and showed that intracellular parasite burden was similar in control and Stat1−/− PEMs for the first 48 h of infection despite the difference in phagosomal pH during this time period (Figure 7). Both survival of wild-type and attenuated *lpg* ^−^ mutant parasites was equally enhanced in Stat1−/− PEMs between day 2 and day 5 post-infection (Figures 2A and 7B), suggesting that the pH-dependent activity compromised in Stat1−/− PEMs acts independent of LPG and its effects on oxidant resistance or phago-lysosomal fusion.

Acidic pH is maintained in phago-lysosomes by the combined action of v-ATPases that transport protons across the membrane, and chloride channels that neutralize the transmembrane potential by counter ion conductivity. Stat1−/− PEMs were normal in phagosome maturation as judged by the kinetics of phago-lysosomal fusion and the accumulation of the late endosomal marker protein rab7 in the mutant phagosomes (Figure 4A and 4B). Dissociation of the molecular events required for vesicular acidification in Stat1−/− cells by ratio-fluorescence measurements indicated functional vATPase activity (Figure 5), which was limited by the increasing transmembrane potential during proton transport and a selective defect in charge neutralization (Figure 6). The mechanism how Stat1 regulates counter-ion conductivity remains elusive and is currently under investigation. Possible mechanisms include a direct transcriptional activation of chloride channel expression or indirect effects on expression of regulatory molecules that modify chloride channel activities, such as p53 [47], erk7 [48] or c-Src [49].

In summary, our data provide evidence for a novel IFNγ-independent function of Stat1 in phagosome acidification, which may have important implications for the interpretation of data previously obtained by others in Stat1-deficient animals. For example, Stat1−/− mice have been recently shown to display an unexpected increase in bone mass, which was attributed to a dysregulation of osteoclast differentiation [50]. Bone remodeling occurs by terminally differentiated cells of the monocyte-macrophage lineage termed osteoclasts, which generate an acidic compartment on the surface of the bone required for resorption ([26] and references therein). Conceivably, a defect in Stat1−/− osteoclast in vesicular acidification similar to the one we describe here for Stat1−/− macrophages may have a major impact on bone homeostasis and thus may substantially participate in increased bone formation observed in these mice. More significantly, Stat1−/− mice were widely used to study the role of IFNγ-mediated immunity to various pathogens. Given the importance of vesicular pH in either resistance to bacterial and protozoan pathogens, and its relevance in the uncoating process during viral entry, the role of constitutive Stat1 activity in innate anti-microbial resistance may have to be re-investigated in light of its potential role in acidification.

## Materials and Methods

### Mice and parasites

129/Sv control mice and mice inactivated for Stat1 expression (referred to as Stat1−/− or Stat1-deficient, [11]) were purchased from Taconic (Germantown, NY). All animals were handled in strict accordance with good animal practice as defined by the relevant national and/or local animal welfare bodies, and all animal work was approved by the appropriate institutional committee. *Leishmania major* strain LV39clone5 (Rho/SU/59/P, [51]) was grown in M199 medium at 26°C as previously described [52]. The LPG-deficient *lpg1* ^−^ *null* mutant was maintained in media supplemented with 16 µg/ml hygromycin B and 20 µM puromycin as described [17]. Axenic amastigotes of *L. donovani* (strain LD1SR, [53]) were cultured at 37°C in M199 supplemented with 20% FCS at pH 5.5 according to Zilberstein et al. [54].

### Mouse and macrophage infection

Virulence was assessed following inoculation of 10^6^ promastigote parasites from day 4 of stationary culture into the footpad of 6 to 8 weeks old female Stat1−/− mice and congenic SV129 controls. Infections were monitored by comparing the thickness of the injected and uninjected footpads with a Vernier caliper. Murine bone marrow macrophages (BMM) were obtained from the femurs of female mice and differentiated in vitro in the presence of M-CSF as described [55]. Peritoneal exudate macrophages (PEM) were elicited by injection of 2 ml endotoxin-free starch suspension (2% w/v in normal saline) into mice. Cells were isolated three days later by peritoneal lavage using cold Dulbecco's modified Eagles medium (DMEM), washed and resuspended in DMEM/10% FBS. For infection, PEM were seeded in 12 well plates onto 18 mm glass cover slips (3×10^5^ cells/ml) and non adherent cells were removed by washing after 30 min incubation at 37°C in 5% CO_2_. Adherent PEM were infected with complement-opsonized promastigotes from day 4 of stationary growth [56] or lesion-derived amastigotes at a multiplicity of infection of 10 parasites per macrophage. Following 2 hours incubation at 33°C in DMEM 0.7% BSA under serum free conditions, non-phagocytosed parasites were removed by multiple washing steps with DMEM without FBS and incubation was proceeded for another 5 days at 33°C. Growth of extracellular parasites was prevented during this period by washing the cells once a day. The number of intracellular parasites was monitored at 2 h, 24 h, 48 h and120 h post-infection by nuclear staining and fluorescence microscopy as described [17]. All culture media were tested to be endotoxin-free using the Pyrotell LAL test kit (Associates of Cape Cod Inc., MA).

### Determination of superoxide, nitric oxide, and IL-12

Superoxide was measured by the ferricytochrome reduction assay [57]. PEMs were washed with Hank's buffered saline solution (HBSS), and incubated for 90 min at 37°C with zymosan (10 particles per cell), purified metacyclic WT (MOI = 10) or *lpg1* ^−^ promastigotes (MOI = 3) in 80 µM ferricytochrome c/HBSS. Supernatants were cleared by centrifugation at 4° and the concentration of reduced cytochrome *c* was determined spectrophotometrically at 550 nm (ε_550 nm_ = 2.1×10^4^ M^−1^ cm^−1^). The background was determined in equally treated control cells in the presence of 100 ng/ml superoxide dismutase (Sigma) in Hank's Balanced Salt Solution (HBSS). NO-derived nitrite in culture supernatants was determined by the Griess reaction [58]. Briefly, 100 µl were removed from conditioned medium, incubated with an equal volume of Griess reagent (1% sulfanilamide/0.1% naphthyl ethylene diamine dihydrochloride/2.5% H_3_PO_4_) at room temperature for 10 min, and the NO_2_^−^ concentration was determined in spectrophotometrically a at 550 nm using NaNO_2_ as a standard. IL-12 (p40) levels were determined in the PEM culture supernatants by an ELISA capture method (Pharmingen, San Diego, CA). Briefly, microtiter plates coated with a capture monoclonal anti-IL-12p40 antibody were incubated with 100 µl of culture supernatant, and bound IL-12 was detected with polyclonal rabbit anti-IL12p40 antibody and peroxidase-conjugated sheep anti-rabbit antibody.

### Immunfluorescence staining

Cells were washed once in phosphate buffered saline (PBS), permeabilized with 100% methanol (−20°C) for 30 seconds and re-hydrated for 10 min at RT in PBS. Preparations were sequentially incubated for 20 min at 37°C with 1/100 dilutions of rab7 primary antibody (Santa Cruz, CA) and 1/100 dilution of FITC conjugated anti-rabbit secondary antibodies as described [59].

### Surface labeling

Zymosan particles or amastigote parasites were labeled for 20 min at 4°C with NHS-carboxyfluoresceine (250 µg/ml, Boehringer Mannheim, Germany) or Succinate-Texas Red (Molecular Probes, OR) in 100 mM NaHCO_3_, 150 mM NaCl at pH 7.6, and washed three times in serum-free DMEM by centrifugation at 1000×g for 5 min.

### Phagolysosomal fusion

PEM were seeded in 12 well plates onto 18 mm glass cover slips (3×10^5^ cells/ml), and incubated overnight (at least 12 h) in DMEM supplemented with 10% FCS and 2.5 mg/ml FITC-conjugated dextran (10 kD, lysine fixable, Molecular Probes, OR). Cells were washed vigorously and incubated at 4° for 20 min with stationary-phase promastigote parasites at a multiplicity of infection (MOI) of 10 parasites per host cell. were infected for 2 h at 33° at for WT or synchronous parasite uptake was achieved For synchronous infections, parasites were incubated to allow attachment, Free parasites were removed by washing, and synchronous infection was achieved by temperature shift to 37°C [60]. Fusogenic FITC-positive phagosomes were quantified by fluorescence microscopy on paraformaldehyde-fixed preparations over a period of 3 hrs following uptake.

### Measurement of intracellular pH

All pH measurements were performed *in situ* with conjugates of fluorescein isothiocyanate. The pH response of the conjugated dye was calibrated in solution and in cells where intracellular compartments were equilibrated with medium pH as described previously [19].

### Phagosomal pH

Monolayers of peritoneal macrophages were incubated with fluorescein-conjugated parasites or zymosan particles for 30 min at 37°C in a humidified CO_2_ incubator (ratio ca. 10 particles or parasites per macrophage). Cells were washed rigorously, incubated further for 2 h at 37°C and phagosomal pH was assessed in an Aminco SPF-500 spectrofluorimeter as previously described [61]. Parasite- and zymosan-conjugates were calibrated in each of the cells employed in these studies (not shown). The pKs of the free dye and dye conjugates were identical in solution and for intracellular measurements indicating that they were reporting the vesicle pH and not conditions particular to the particle surface, compartment or dye conjugate [62],[63]. These measurements were used to determine vesicle pH in the following studies.

### Cytoplasmic pH

Cells were incubated in 10 µM in 2′,7′-bis(2carboxyethyl)-5-carboxyfluoresceine-tetraacetoxymethyl ester, BCECF-AM (Molecular Probes, OR), for 30 min and washed as previously described [64]. Intracellular cytoplasmic fluorescence was calibrated, and intracellular pK and pH response were determined using buffered Nigericin solutions [61],[64].

### Endosomal pH

Macrophage monolayers were incubated overnight (at least 12 h) in DMEM supplemented with 10% FCS and 2.5 mg/ml FITC-conjugated dextran (10 kD, lysine fixable, from Molecular Probes, OR). Cells were washed vigorously and endo-lysosomal pH was assessed by ratio-fluorescence determination. The buffering capacity was determined as described [21].

### pH measurement on isolated phagosomes

Macrophage monolayers were allowed to phagocytose FITC-conjugated zymosan, collected by scraping in turtle buffer supplemented with 1 mM dithiothreitol [26] and disrupted in a tight fitting Dounce homogenizer. Undisrupted cells and debris were removed by centrifugation at 1500×g for 5 min and the phagosomes sedimented at 10000×g. The pellet was suspended in 140 mM KCl, 10 mM HEPES pH 7.0, and acidification of the vesicles was initiated by the addition of 2.5 mM potassium ATP and 1 mM MgSO_4_. The intravesicular pH was assessed using ratio-fluorescence determinations following calibration of intra-phagosomal pH with Nigericin [61],[64].

### Acridine orange fluorescence quenching

Isolated phagosomes containing unlabeled zymosan were disrupted by one freeze-thaw cycle at −70°C, zymosan particles were removed by centrifugation at 2000×g and the membrane fraction was pelleted at 100000×g for 60 min at 4°C. The assay was performed as described [26].

### Western blot

Western blot analysis was performed using the enhanced chemiluminescence (ECL) detection kit from Amersham International, UK. Cellular extracts were resolved by SDS-PAGE and electroblotted onto nitro-cellulose membrane (Amersham International, UK). Antibody incubation and detection were performed according to the protocol supplied with the kit. Primary antibody AB656 [26] was diluted 1/200.
